# MSL1 Promotes Liver Regeneration by Driving Phase Separation of STAT3 and Histone H4 and Enhancing Their Acetylation

**DOI:** 10.1002/advs.202301094

**Published:** 2023-06-06

**Authors:** Yucheng He, Shichao Wang, Shenghui Liu, Dan Qin, Zhangmei Liu, Liqiang Wang, Xiangmei Chen, Lisheng Zhang

**Affiliations:** ^1^ College of Veterinary Medicine/Bio‐medical Center Huazhong Agricultural University Wuhan Hubei 430070 China; ^2^ Department of Nephrology Chinese PLA General Hospital Chinese PLA Institute of Nephrology State Key Laboratory of Kidney Diseases National Clinical Research Center for Kidney Diseases 28th Fuxing Road Beijing 100853 China

**Keywords:** acetyl coenzyme A, histone H4, liquid–liquid phase separation, liver regeneration, MSL1, STAT3

## Abstract

Male‐specific lethal 1 (MSL1) is critical for the formation of MSL histone acetyltransferase complex which acetylates histone H4 Lys16 (H4K16ac) to activate gene expression. However, the role of MSL1 in liver regeneration is poorly understood. Here, this work identifies MSL1 as a key regulator of STAT3 and histone H4 (H4) in hepatocytes. MSL1 forms condensates with STAT3 or H4 through liquid–liquid phase separation to enrich acetyl‐coenzyme A (Ac‐CoA), and Ac‐CoA in turn enhances MSL1 condensate formation, synergetically promoting the acetylation of STAT3 K685 and H4K16, thus stimulating liver regeneration after partial hepatectomy (PH). Additionally, increasing Ac‐CoA level can enhance STAT3 and H4 acetylation, thus promoting liver regeneration in aged mice. The results demonstrate that MSL1 condensate‐mediated STAT3 and H4 acetylation play an important role in liver regeneration. Thus, promoting the phase separation of MSL1 and increasing Ac‐CoA level may be a novel therapeutic strategy for acute liver diseases and transplantation.

## Graphical abstract

Liver regeneration is important for the surgical treatment of liver diseases, such as the removal of liver tumors and transplantation. This study reveals that acetyl‐coenzyme A (Ac‐CoA) enhances the phase separation of male‐specific lethal 1 (MSL1), and MSL1 promotes liver regeneration after partial hepatectomy in a phase separation‐dependent manner. Targeting this pathway may be a promising therapeutic approach for liver regeneration.

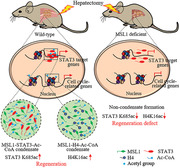

## 1 Introduction

The adult liver has a strong regeneration capacity after injury, and hepatocytes proliferate rapidly in a highly synchronized manner in response to the liver injury caused by surgical removal, chemical drug, or infection.^[^ ^1^ ^]^ The understanding of the regulatory mechanisms underlying liver regeneration is of vital importance for developing novel strategies to enhance the regenerative process and prevent acute liver failure. Histone acetylation and non‐histone protein acetylation influence multiple cellular, physiological, and pathological processes including the liver injury repair.^[^ ^2^ ^]^ However, the specific roles of acetylation in liver regeneration remain largely unclear.

As a scaffold for MSL complex, male‐specific lethal 1 (MSL1) uses short interacting peptides for the recruitment of MSL3 and males absent on the frist (MOF) into the MSL complex, and MSL1 is essential to maintain its acetyltransferase activity of this complex, which specifically acetylates histone H4 Lys16 (H4K16ac).^[^ ^3^ ^]^ In *Drosophila*, the MSL complex consists of five proteins (MSL1, MSL2, MSL3, MLE, and MOF) and two long noncoding RNAs (roX1 and roX2), and this complex can increase expression of X‐linked genes by 2 folds in male flies through H4K16ac.^[^ ^4^ ^]^ The MSL1 of mouse and *Drosophila* consists of 616 and 1039 amino acid residues, respectively, with similar domains.^[^ ^3^ ^]^ Although no RNA has been identified from MSL complex in mammals, at least four MSL proteins (MSL1, MSL2, MSL3, and MOF) were identified from this complex.^[^ ^5^ ^]^ H4K16ac can increase chromatin accessibility to promote gene expression,^[^ ^6^ ^]^ and it plays a role in cell cycle progression and cell proliferation.^[^ ^7^ ^]^ Nevertheless, whether H4K16ac regulates liver regeneration remains unknown.

Priming of hepatocytes and rapidly reentry into the cell cycle after partial hepatectomy (PH) are critical for liver regeneration.^[^ ^8^ ^]^ Signal transducer and activator of transcription 3 (STAT3) is a key transcriptional mediator for priming of hepatocyte, and it plays an essential role in cell proliferation and liver regeneration in response to interleukin 6 (IL‐6) stimulation.^[^ ^9^ ^]^ Posttranslational modifications of STAT3 in response to cytokine treatment mainly include phosphorylation on tyrosine 705.^[^ ^10^ ^]^ STAT3 acetylation can also respond to cytokine treatment, and promotes STAT3 phosphorylation and transcriptional activation.^[^ ^11^, ^12^ ^]^ In cytokine‐treated splenocytes, STAT3 acetylation is positively correlated with its phosphorylation.^[^ ^13^ ^]^ and in fasted mice, STAT3 acetylation promotes its phosphorylation.^[^ ^11^ ^]^ MOF, the catalytic subunit of the MSL complex, is involved in the acetylation of a variety of non‐histone proteins.^[^ ^14^ ^]^ However, whether the MSL complex is involved in the acetylation of STAT3 remains unclear.

Phase separation can compartmentalize proteins, nucleic acids, or small molecules to form condensates, and phase separation is widely involved in the regulation of cellular signaling.^[^ ^15^ ^]^ Proteins with a large number of intrinsically disordered regions (IDRs) are more prone to phase separation.^[^ ^16^ ^]^ Phase separation can increase enzymatic reaction rates by increasing concentrations of enzymes and substrates (mass action).^[^ ^17^ ^]^ ATP can not only be involved in the regulation of phase separation,^[^ ^18^, ^19^ ^]^ but also can be enriched in phase‐separated condensates as a substrate.^[^ ^20^ ^]^ Acetyl‐coenzyme A (Ac‐CoA), as a central metabolite, has a similar structure to ATP, but it is not clear whether Ac‐CoA has the same function as ATP. Recent studies have shown that liquid–liquid phase separation can control protein acetylation and cell cycle.^[^ ^21^, ^22^ ^]^ Nevertheless, whether phase separation regulates liver regeneration remains unknown.

In addition to its prominent role in metabolism and biosynthesis, Ac‐CoA provides acetyl groups for protein acetylation.^[^ ^23^ ^]^ Acetylation is directly related to Ac‐CoA level, and cell compartment‐specific generation of Ac‐CoA can locally drive acetylation.^[^ ^24^ ^]^ Ac‐CoA has anti‐aging effects by regulating epigenetic modifications.^[^ ^25^ ^]^ Many of Ac‐CoA‐sensitive genes are involved in the cell cycle, DNA replication, and cell adhesion, and migration,^[^ ^26^ ^]^ suggesting that Ac‐CoA may promote liver regeneration. As an Ac‐CoA carboxylase inhibitor, GS‐0976 can increase hepatic Ac‐CoA level and alleviate nonalcoholic fatty liver disease in rats,^[^ ^27^ ^]^ but it is unclear whether Ac‐CoA level is corrected with liver regeneration after acute liver injury. Here, we provide experimental evidence that Ac‐CoA enhanced phase separation of MSL1, and the MSL1 promoted hepatocyte proliferation in a phase separation‐dependent manner. In addition, increasing Ac‐CoA levels enhanced liver regeneration in aged mice.

## 2 Results

### 2.1 Deletion of MSL1 Impairs Liver Regeneration After PH

**Figure 1:**
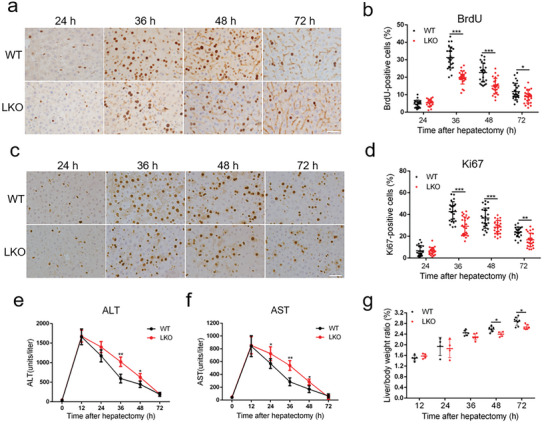
Loss of MSL1 impairs liver regeneration after partial hepatectomy (PH). a) BrdU staining of liver tissues from WT and LKO mice at 24–72 h after PH. Scale bar, 50 µm. b) BrdU‐positive cell count at 24–72 h after PH, *n* = 4–5 mice per group, 5 fields (215 × 325 µm^2^) quantified/animal. c) Ki67 staining of liver tissues from WT and LKO mice at 24–72 h after PH. Scale bar, 50 µm. d) Ki67‐positive cell count at 24–72 h after PH, *n* = 4–5 mice per group, 5 fields (215 × 325 µm^2^) quantified/animal. e,f) Serum alanine aminotransferase (ALT) (e) and aspartate aminotransferase (AST) (f) levels in WT and LKO mice at 24–72 h after PH (*n* = 4 mice per group). g) Liver‐to‐body weight ratio of WT and LKO mice after PH (*n* = 4–7 mice per group). The data were expressed as means ± SD. Significant difference was presented at the level of ^*^ *p* < 0.05, ^**^ *p* < 0.01, and ^***^ *p* < 0.001 by two‐tailed Student's *t*‐test.

In contrast to the male lethal phenotype caused by MSL1 mutation in flies,^[^ ^28^ ^]^ Alb‐Cre‐mediated gene deletion did not induce discernible abnormalities in the mouse liver (Figure S1a, Supporting Information). To examine the effect of MSL1 on liver regeneration, MSL1 LKO mice and their WT littermates were subjected to two‐thirds PH.^[^ ^8^ ^]^ Immunoblot and qPCR results confirmed the successful deletion of MSL1 in the liver from LKO mice (Figure S1b,c, Supporting Information). BrdU and Ki67 staining showed that there were more proliferating hepatocytes in the WT mice than that in the LKO mice (**Figure** **1**a–d). Compared with WT mice, LKO mice exhibited higher levels plasma alanine aminotransferase (ALT) and aspartate aminotransferase (AST) after PH (Figure 1e,f), suggesting that MSL1 deletion aggravated liver injury. Additionally, LKO mice displayed a low liver‐to‐body weight ratio after PH (Figure 1g), indicating attenuated liver mass recovery. These results suggested MSL1 might be an essential factor in liver regeneration.

### 2.2 MSL1 Enhances STAT3 Transcriptional Activity by Interacting with STAT3 During Liver Regeneration

**Figure 2:**
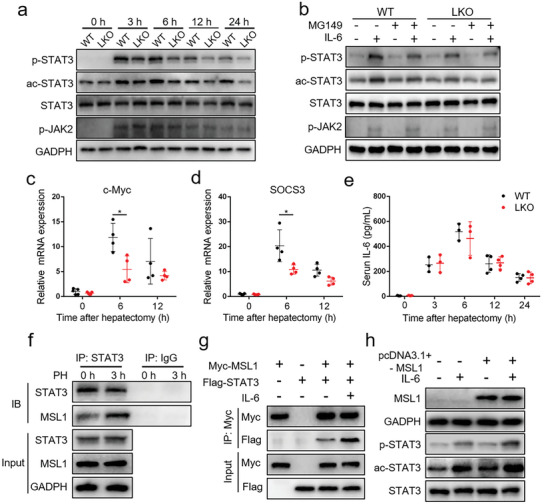
MSL1 enhances STAT3 transcriptional activity by interacting with STAT3 during liver regeneration. a) Immunoblot analysis of liver tissue lysates from WT and LKO mice at 24–72 h after partial hepatectomy (PH) using the indicated antibodies. b) Immunoblot analysis of primary mouse hepatocytes from WT and LKO mice treated with 50 × 10^−6^ m MG149 for 24 h with or without 30‐min 10 ng mL^−1^ IL‐6 treatment using the indicated antibodies. c,d) qRT‐PCR analysis of c‐Myc (c) and SOCS3 (d) mRNA expression in the regenerating liver (*n* = 4–5 mice per group). e) ELISA analysis of serum IL‐6 level in WT and LKO mice at 24–72 h after PH (*n* = 3–4 mice per group). f) Coimmunoprecipitation analysis of MSL1 and STAT3 in liver tissues from WT mice. g) HEK293T cells were transfected with Myc‐MSL1‐encoding plasmids and with or without Flag‐STAT3 plasmids, followed by culturing for 24 h without or with 10 ng mL^−1^ IL‐6 for 30 min. Coimmunoprecipitation analysis was performed. h) Hep1‐6 cells were transfected with plasmids for MSL1 or control vector for 24 h and treated without or with 10 ng mL^−1^ IL‐6 for 30 min. Immunoblot analysis using the indicated antibodies. The data were expressed as means ± SD. Significant difference was presented at the level of *p < 0.05 by two‐tailed Student's *t*‐test.

Given the critical role of the IL‐6–STAT3 signaling pathway in the initiation of liver regeneration after PH,^[^ ^29^ ^]^ we examined whether MSL1 deficiency affected STAT3 activation or not. At 3, 6, 12, and 24 h post PH, the acetylation of STAT3 K685 (ac‐STAT3) in the livers was lower in LKO mice than that in WT mice (**Figure** **2**a and Figure S2a, Supporting Information). STAT3 Y705 phosphorylation (p‐STAT3) level was positively correlated with ac‐STAT3 level (Figure 2a and Figure S2b, Supporting Information), indicating that p‐STAT3 and ac‐STAT3 might be functionally relevant. Furthermore, ac‐STAT3 and p‐STAT3 levels induced by IL‐6 were reduced in MSL1‐deficiency primary hepatocytes, and MG149 (a specific MOF inhibitor) treatment inhibited ac‐STAT3 and p‐STAT3 in WT primary hepatocytes (Figure 2b and Figure S2d,e, Supporting Information), indicating that MSL1‐MOF might participate in acetylation and phosphorylation of STAT3. Consistently, expression levels of STAT3 target genes including c‐Myc and SOCS3 were decreased in the liver of LKO mice after PH (Figure 2c,d). Interestingly, LKO and WT mice had similar elevation in serum IL‐6 level at various time points after PH (Figure 2e), and STAT3 acetylation and phosphorylation were inhibited in LKO mouse liver and primary hepatocytes in kinase JAK2‐independent manner (Figure 2a,b and Figure S2c,f, Supporting Information), suggesting that the augmentation of STAT3 phosphorylation level might be due to STAT3 acetylation by MSL1.

Acetylation is directly related to Ac‐CoA levels.^[^ ^24^ ^]^ CMS‐121 (Ac‐CoA carboxylase 1 inhibitor) and SB 204 990 (ATP citrate lyase inhibitor) can increase and decrease Ac‐CoA level in cells, respectively (Figure S2g, Supporting Information).^[^ ^25^, ^30^ ^]^ In IL‐6‐induced WT primary hepatocytes, CMS‐12 increased ac‐STAT3 and p‐STAT3 levels, whereas SB 204 990 decreased their levels. However, the effects of CMS‐121 and SB 204 990 on ac‐STAT3 and p‐STAT3 levels were weakened in MSL1 deficiency primary hepatocytes (Figure S2h,i, Supporting Information), suggesting that Ac‐CoA partially participated in the regulation of ac‐STAT3 and p‐STAT3 through MSL1.

Both MSL1 and STAT3 contain a coiled‐coil (CC) domain, and the CC domain plays an important role in mediating protein interaction.^[^ ^31^ ^]^ Considering this, we examined whether the MSL1 and STAT3 proteins interacted each other. STAT3 and MSL1 proteins coimmunoprecipitated in vivo, and exogenous proteins Myc‐MSL1 and Flag‐STAT3 interacted, and the activation of STAT3 enhanced the interaction in vivo and in vitro (Figure 2f,g and Figure S2j,k, Supporting Information). Overexpression of MSL1 promoted acetylation and phosphorylation of STAT3 in IL‐6‐induced Hep1‐6 cells (Figure 2h and Figure S2l, Supporting Information). These data demonstrated that MSL1 interacted with STAT3, thus enhancing STAT3 activity and its downstream gene expression.

### 2.3 MSL1 Regulates Cell Cycle‐Related Gene Expression Through H4K16ac During Liver Regeneration

**Figure 3:**
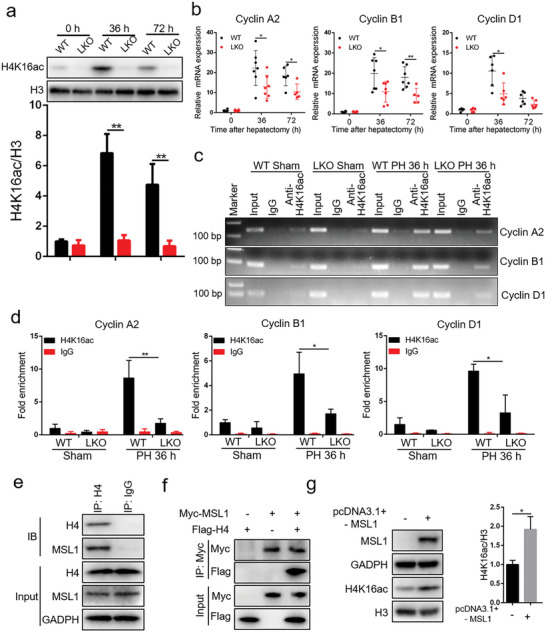
MSL1 regulates cell cycle genes through H4K16 acetylation. a) Immunoblot analysis of liver tissues lysates prepared from WT and LKO mice at 36 and 72 h after partial hepatectomy (PH) using the indicated antibodies. Lower panel show the quantification of protein levels H4K16ac by densitometric analysis and normalization versus loading control (*n* = 3). b) qRT‐PCR analysis of Cyclin A2, Cyclin B1 and Cyclin D1 mRNA expression in the regenerating liver (*n* = 5–7 mice per group). c,d) ChIP‐PCR (c) and ChIP‐qPCR (d) assay of H4 K16ac level on Cyclin A2, Cyclin B1, and Cyclin D1 promoters using chromatin solutions prepared from WT and LKO mice at 36 h post PH or the sham procedure (*n* = 3 mice per group). e) Coimmunoprecipitation analysis of MSL1 and H4 in liver tissues from WT mice. f) HEK293T cells were transfected with Myc‐MSL1‐encoding plasmids and with or without Flag‐H4 plasmids, followed by culturing for 24 h. Coimmunoprecipitation analysis was performed. g) Hep1‐6 cells were transfected with plasmids for MSL1 or control vector for 24 h. Immunoblot analysis using the indicated antibodies. Right panel show the quantification of protein levels H4K16ac by densitometric analysis and normalization versus loading control (*n* = 3). The data were expressed as means ± SD. Significant difference was presented at the level of ^*^ *p* < 0.05 and ^**^ *p* < 0.01 by two‐tailed Student's *t*‐test.

The enrichment of H4K16ac (a major target of the MSL complex) on the promoters activates gene expression by regulating nucleosome accessibility.^[^ ^5^, ^6^ ^]^ We found that compared with that in WT mice, H4K16ac level was significantly decreased in LKO mice after PH (**Figure** **3**a). The previous ChIP‐seq data showed that H4K16ac was enriched on cell cycle‐related gene promoters.^[^ ^32^ ^]^ Further, we tested whether reduction of H4K16ac level in LKO mice after PH altered the expression of cell cycle‐related genes. As shown in Figure 3b, the expressions of cell cycle‐related genes cyclin A2, cyclin B1, and cyclin D1 were reduced in the LKO mice at 36 and 72 h post PH. Our ChIP analysis showed that the deletion of MSL1 compromised the increase in H4K16ac at the cyclin A2, cyclin B1 and cyclin D1 promoters after PH (Figure 3c,d). Similar to STAT3, histone H4 (H4) and MSL1 proteins coimmunoprecipitated in vivo (Figure 3e), exogenous proteins Myc‐MSL1 and Flag‐H4 interacted (Figure 3f), and overexpression of MSL1 promoted H4K16 acetylation in Hep1‐6 cells (Figure 3g). These results indicated that in regenerating liver, MSL1 was needed for the H4K16 hyperacetylation associated with an increasing expression of the cyclin genes.

### 2.4 Overexpression of MSL1 in the Hepatocytes Promoted STAT3 and H4K16 Acetylation

To further explore the effect of MSL1 on the acetylation of STAT3 and H4, hydrodynamic tail vein delivery of GFP or GFP‐MSL1 expressed plasmids into mice to result in approximately 10% of hepatocytes overexpressing GFP or GFP‐MSL1 in the liver. Primary mouse hepatocytes were isolated at 3 or 36 h after PH, and GFP‐positive cells were sorted by fluorescence activated cell sorting (FACS). We found that at 3 h post PH, the levels of p‐STAT3 and ac‐STAT3 in GFP‐MSL1‐overexpressing hepatocytes were significantly increased compared with control hepatocytes (Figure S3a,b, Supporting Information), and expression levels of STAT3 target genes including c‐Myc and SOCS3 were increased in GFP‐MSL1‐overexpressing hepatocytes (Figure S3c, Supporting Information). Similarly, at 36 h post PH, compared with control hepatocytes, H4K16ac level was significantly increased in GFP‐MSL1‐overexpressing hepatocytes (Figure S3d,e, Supporting Information), the expressions of cell cycle‐related genes cyclin A2, cyclin B1, and cyclin D1 were increased in GFP‐MSL1‐overexpressing hepatocytes (Figure S3f, Supporting Information). These results indicated that overexpression of MSL1 promoted the acetylation of STAT3 and H4K16 and their downstream gene expression in regenerating liver.

### 2.5 Ac‐CoA Modulates Phase Separation of MSL1 in Cells and In Vitro

**Figure 4:**
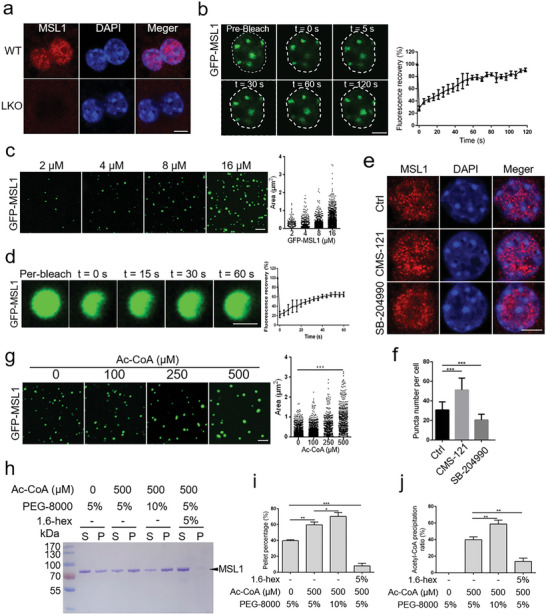
MSL1 phase separation modulated by acetyl‐CoA in cells and in vitro. a) Primary mouse hepatocytes immunostained with an MSL1 antibody in WT and LKO mice. Scale bar, 5 µm. b) Fluorescence recovery after photo bleach (FRAP) time‐lapse images and quantification of GFP‐MSL1 condensates in live transfected HEK293T cells. Data are representative of three independent FRAP events. Scale bar, 5 µm. c) Confocal images and quantification of GFP‐MSL1 condensates at increasing protein concentrations (2 × 10^−6^–16 × 10^−6^ m). *n* = 3 fields (100 × 100 µm^2^). Scale bar, 5 µm. d) FRAP assays and quantification of GFP‐MSL1 (8 × 10^−6^ m) condensates. Data are representative of three independent FRAP events. Scale bar, 1 µm. e) Immunofluorescence images of MSL1 in primary hepatocytes treated with CMS‐121 (1 × 10^−6^ m) or SB‐204990 (10 × 10^−6^ m) for 24 h. Scale bar, 5 µm. f) Quantification of MSL1 nuclear puncta in primary hepatocytes (*n* = 20 cells). g) Confocal images of GFP‐MSL1 condensates (10 × 10^−6^ m) at increasing acetyl‐coenzyme A (Ac‐CoA) concentrations (0–500 × 10^−6^ m). *n* = 3 fields (100 × 100 µm^2^). Scale bar, 5 µm. h) SDS–PAGE assay of MSL1 recovered from the aqueous phase or supernatant (S) and the condensed phase or pellet (P). Ac‐CoA, PEG‐8000, or 1.6‐hex were added at the indicated concentrations. i) Quantification of the protein fraction recovered from pellets in the sedimentation assays. The sedimentation experiments were conducted in triplicates. j) Quantification of the Ac‐CoA recovered from pellets in the sedimentation assays, Ac‐CoA, PEG‐8000, and 1.6‐hex were added at the indicated concentrations. The sedimentation experiments were conducted in triplicates. The data were expressed as means ± SD. Significant difference was presented at the level of ^*^ *p* < 0.05, ^**^ *p* <0.01, and ^***^ *p* <0.001 by two‐tailed Student's *t*‐test.

MSL1 contains large IDRs (Figure S4a, Supporting Information). Given that IDRs play important roles in promoting phase separation of proteins,^[^ ^33^ ^]^ we next investigated whether MSL1 underwent phase separation. Immunofluorescence showed the formation of nuclear puncta of MSL1 in primary hepatocytes (**Figure** **4**a). Ectopic expression illustrated that GFP‐MSL1 proteins formed spherical condensates in the nucleus, but these condensates could be disrupted by 5% 1,6‐hexanediol (1,6‐hex) (Figure S4b,c, Supporting Information). The fluorescence signal of GFP‐MSL1 condensates in the HEK 293T cells exhibited fast recovery after photo bleaching (Figure 4b), indicating that MSL1 condensates had liquid‐like properties. To identify the domain responsible for phase separation of MSL1, we mutated MSL1 and found that there was no longer phase separation of MSL1 after the CC domain or the IDR3 region was removed (Figure S4d,e, Supporting Information). Further, we examined the potential phase‐separation capacity of MSL1 in vitro, expressed the recombinant GFP‐MSL1 fusion proteins, and purified them from *Escherichia coli* (*E. coli*) (Figure S4f, Supporting Information). After 10 × 10^−3^ m GFP‐MSL1 was added into the phase separation buffer (20 × 10^−3^ m HEPES, 100 × 10^−3^ m NaCl, 5% PEG8000, pH 7.8), the solution became turbid, and droplets was formed under light microscopy, whereas the solution added with GFP was relatively clear (Figure S4g, Supporting Information). Confocal images showed that purified GFP‐MSL1 formed microsized condensates in solutions, and the condensates became larger in size and in number with the increasing GFP‐MSL1 concentrations (Figure 4c). Salt (NaCl) and 1,6‐hex inhibited the formation of GFP‐MSL1 condensates, whereas PEG‐8000 promoted their formation (Figure S4h–j, Supporting Information), and the fluorescence signal of these condensates exhibited fast recovery after photo bleaching (Figure 4d), confirming the liquid‐like properties of GFP‐MSL1 condensates.

Ac‐CoA serves as an essential second messenger and the sole donor of acetyl groups for acetyltransferases,^[^ ^23^ ^]^ and Ac‐CoA has the structure partially similar to ATP involved in the regulation of phase separation.^[^ ^18^ ^]^ Considering this, we wondered whether Ac‐CoA could affect the phase separation of MSL1. The formed MSL1 nuclear puncta in cells increased after CMS‐121 treatment, but decreased after SB‐204990 treatment (Figure 4e,f), indicating that Ac‐CoA promoted the phase separation of MSL1. The in vitro experiment also showed that AC‐CoA promoted phase separation of GFP‐MSL1 (Figure 4g). ATP can be enriched in phase‐separated condensates,^[^ ^20^ ^]^ based on which we speculated that Ac‐CoA might have similar enrichment property in MSL1 condensates. To test our speculation, we performed a sedimentation assay to separate the condensed liquid phase from the aqueous solutions by centrifugation and assessed protein and Ac‐CoA levels in each phase (Figure S4k, Supporting Information). Nearly half of MSL1 protein was recovered from the condensed liquid phase. The concentration increase of PEG‐8000 or the addition of Ac‐CoA promoted the recovery of the condensate, while the addition of 1.6‐hex inhibited the recovery of the condensate (Figure 4h,i). Importantly, with the increased MSL1 condensate recovery, the Ac‐CoA level was increased (Figure 4j), indicating that Ac‐CoA was enriched in MSL1 condensates. Taken together, these data demonstrated that MSL1 could phase separate in cells and in vitro, and that Ac‐CoA could be enriched in MSL1 condensates and promote the phase separation of MSL1.

### 2.6 MSL1 Promotes the Acetylation of STAT3 and H4 by Compartmentalizing STAT3 and H4 in the Phase‐Separation Condensates

**Figure 5:**
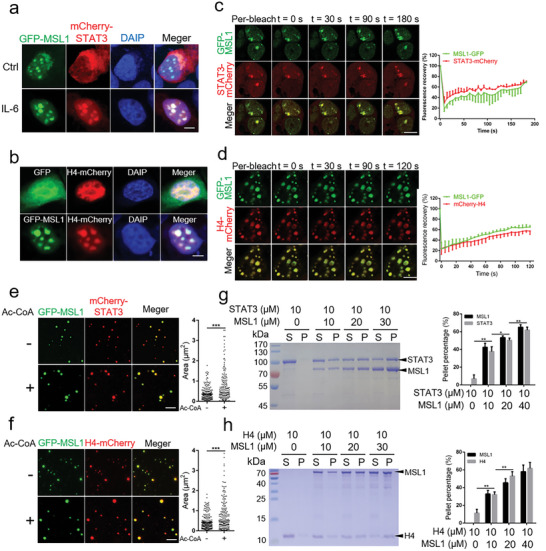
MSL1 compartmentalizes STAT3 or H4 in phase‐separation condensates to promote the acetylation of STAT3 or H4. a) Confocal images of condensates in fixed HEK293T cells transfected with GFP‐MSL1‐ and mCherry‐STAT3‐encoding plasmids for 24 h without or with 10 ng mL^−1^ IL‐6 for 30 min. Scale bar, 5 µm. b) Confocal images of phase condensates in fixed HEK293T cells transfected with H4‐mCherry‐encoding plasmid with GFP‐ or GFP‐MSL1‐encoding plasmid transfection. Scale bar, 5 µm. c,d) Fluorescence recovery after photo bleach (FRAP) assays and quantification of condensates in live HEK293T cells transfected with GFP‐MSL1 and mCherry‐STAT3 (c) or H4‐mCherry (d). Data are representative of three independent FRAP events. Scale bar, 5 µm. e,f) Confocal images of GFP‐MSL1 with mCherry‐STAT3 (e) or H4‐mCherry (f) in vitro with or without acetyl‐coenzyme A (Ac‐CoA) (500 × 10^−6^ m). *n* = 3 fields (60 × 60 µm^2^). Scale bar, 5 µm. g,h) SDS–PAGE assay of MSL1 and STAT3 condensates (g) or MSL1 and H4 condensates (h) recovered from the supernatant (S) and pellet (P). Proteins were added at the indicated concentrations. Quantification results of protein fraction recovered from pellets in the sedimentation assays are presented in the right panel. The sedimentation experiments were conducted in triplicates. The data were expressed as means ± SD. Significant difference was presented at the level of ^*^ *p* < 0.05, ^**^ *p* < 0.01, and ^***^ *p* < 0.001 by two‐tailed Student's *t*‐test.

Since STAT3 and histones can be phase separated in cells or in vitro,^[^ ^34^, ^35^ ^]^ we further investigated whether MSL1 could form condensates with STAT3 or H4. The results showed that after expressing mCherry‐STAT3 alone, mCherry‐STAT3 underwent no phase separation with or without IL‐6 treatment (Figure S5a, Supporting Information), whereas after coexpression of GFP‐MSL1 and mCherry‐STAT3, mCherry‐STAT3 entered the nucleus and was phase separated from GFP‐MSL1 following IL‐6 treatment (**Figure** **5**a). Likewise, H4‐mCherry was distributed in the nucleus without phase separation after coexpression H4‐mCherry and GFP, but H4‐mCherry entered the GFP‐MSL1 condensates after coexpression of GFP‐MSL1 and H4‐mCherry (Figure 5b). The fluorescence signal of GFP‐MSL1‐mCherry‐STAT3 and GFP‐MSL1‐H4‐mCherry condensates exhibited fast recovery after photo bleaching (Figure 5c,d), indicating that condensates formed by MSL1 with STAT3 or H4 had liquid‐like properties.

To examine whether the MSL1 with STAT3 or H4 had the phase‐separation capacity in vitro, recombinant mCherry‐STAT3 and H4‐mCherry fusion proteins were expressed and purified from *E. coli* (Figure S5b,c, Supporting Information). The results showed that mCherry‐STAT3 or H4‐mCherry did not form condensates in phase separation buffer (data not shown), but after GFP‐MSL1 addition, mCherry‐STAT3 or H4‐mCherry formed condensates with GFP‐MSL1, and Ac‐CoA enhanced condensates formation (Figure 5e,f). Similarly, sedimentation analysis showed that STAT3 and H4 were highly soluble in phase separation buffer without the formation of condensates, and that STAT3 and H4 were enriched in the condensed phase in a concentration‐dependent manner after the addition of MSL1 (Figure 5g,h). Taken together, these data suggested that MSL1 could drive the phase separation of STAT3 or H4 in cells and in vitro.

Colocalization analysis indicated that MSL1 condensates contained STAT3 or H4. We next examined whether MSL1 phase separation was required for the acetylation of STAT3 and H4 using the MSL1‐ΔIDR3 mutant that could not form condensates (Figure S4e, Supporting Information). We found that overexpression of MSL1 significantly increased the acetylation of H4K16, and promoted IL‐6‐induced acetylation and phosphorylation of STAT3 in Hep1‐6 cells, whereas these results were not observed in the MSL1‐ΔIDR3 mutant (Figure S5d,e, Supporting Information), suggesting that MSL1 condensates played an important role in the transcriptional activation of STAT3 and the acetylation of H4K16.

### 2.7 GS‐0976 Improves Liver Regeneration by Increasing Hepatic Ac‐CoA Level and Acetylation of STAT3 and H4 in Aged Mice

**Figure 6:**
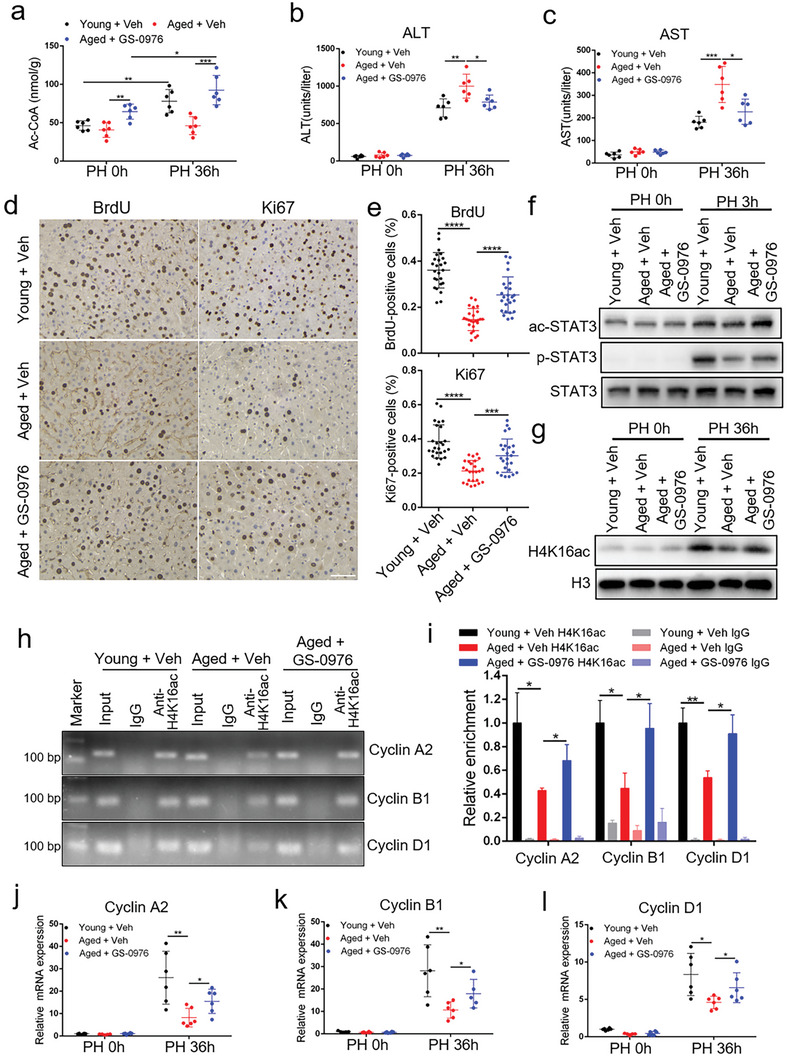
GS‐0976 improves liver regeneration in aged mice by increasing hepatic acetyl‐coenzyme A (Ac‐CoA) levels. a) Hepatic Ac‐CoA level in young mice, aged mice, and aged mice treated with GS‐0976 at 0 and 36 h after partial hepatectomy (PH; *n* = 6 mice per group). b,c) Serum alanine aminotransferase (ALT) (b) and aspartate aminotransferase (AST) (c) levels in young mice, aged mice, and aged mice treated with GS‐0976 at 0 and 36 h after PH (*n* = 6 mice per group). d) BrdU and Ki67 staining of liver tissues from young mice, aged mice, and aged mice treated with GS‐0976 at 36 h after PH. Scale bar, 50 µm. e) BrdU‐ and Ki67‐positive cell count at 36 h after PH, *n* = 5 mice per group, 5 fields (215 × 285 µm^2^) quantified/animal. f,g) Immunoblot analysis of liver tissue lysates from young mice, aged mice, and aged mice treated with GS‐0976 at 0 h, 3 h (f), and 36 h (g) after PH using the indicated antibodies. h,i) ChIP‐PCR (h) and ChIP‐qPCR (i) assays of H4K16ac level on Cyclin A2, Cyclin B1, and Cyclin D1 promoters using chromatin solutions prepared from the livers of young mice, aged mice, and aged mice treated with GS‐0976 at 36 h post PH (*n* = 3 mice per group). j–l) qRT‐PCR analysis of Cyclin A2 (j), Cyclin B1 (k), and Cyclin D1 (l) mRNA expression in the regenerating liver (*n* = 5–6 mice per group). The data were expressed as means ± SD. Significant difference was presented at the level of ^*^ *p* < 0.05, ^**^ *p* < 0.01, ^***^ *p* < 0.001, and ^****^ *p* < 0.0001 by two‐tailed Student's *t*‐test.

The liver regeneration ability of aged mice is attenuated after PH,^[^ ^36^ ^]^ which may be related to the changes of MSL1 expression in the liver of aged mice. We found that MSL1 mRNA and protein levels were decreased in the liver of aged mice (Figure S6a,b, Supporting Information), potentially resulting in the decrease in acetylation of STAT3 and H4K16, eventually impairing liver regeneration in aged mice. Since Ac‐CoA level is positively correlated with protein acetylation levels,^[^ ^37^ ^]^ we further tested whether the elevation of Ac‐CoA level in the livers of aged mice could enhance their liver regeneration. We found that Ac‐CoA level was elevated in the livers of young mice after PH, but not in aged mice, whereas GS‐0976 treatment increased Ac‐CoA level in the livers of aged mice before and after PH (**Figure** **6**a). GS‐0976 treatment decreased the serum ALT and AST levels in aged mice after PH (Figure 6b,c), but increased the number of BrdU‐ and Ki67‐positive cells in the liver (Figure 6d,e). At 3 h post PH, the levels of ac‐STAT3 and p‐STAT3 were decreased in the livers of aged mice relative to young mice, and GS‐0976 treatment alleviated the decrease of ac‐STAT3 and p‐STAT3 levels (Figure 6f and Figure S6c, Supporting Information). Similarly, at 36 h post PH, H4K16ac level was decreased in the livers of aged mice, whereas GS‐0976 treatment alleviated the decrease in H4K16ac level (Figure 6g and Figure S6d, Supporting Information). Correspondingly, GS‐0976 treatment increased H4K16ac level on the promoters of Cyclin A2, B1, and D1 (Figure 6h,i) and upregulated Cyclin A2, B1, and D1 mRNA expression (Figure 6j‐l) in aged mouse liver after PH. These results demonstrated that GS‐0976 might promote liver regeneration in aged mice by increasing hepatic Ac‐CoA levels and promoting acetylation of STAT3 and H4.

To examine whether the GS‐0976 promoted liver regeneration in mice dependent on MSL1, WT and MSL1 LKO mice treatment with GS‐0976. We found that GS‐0976 treatment decreased the serum ALT and AST levels, and increased the number of BrdU‐ and Ki67‐positive hepatocytes in WT mice at 36 h after PH (Figure S6e–g, Supporting Information). However, the effects were weakened in LKO mice (Figure S6e–g, Supporting Information), suggesting that GS‐0976 promoted liver regeneration partially dependent on MSL1.

In summary, these results indicate that hepatocyte‐specific deletion of MSL1 impairs liver regeneration. MSL1 forms condensates with STAT3 or H4 by phase separation to enrich Ac‐CoA and promote the acetylation of STAT3 and H4K16, thus enhancing STAT3 transcriptional activation and up‐regulating the expression of cell cycle‐related genes, ultimately promoting hepatocyte proliferation. Moreover, Ac‐CoA can enhance the phase separation of MSL1, and GS‐0976 treatment can elevate Ac‐CoA level, thus promoting liver regeneration of aged mice.

## 3 Discussion

Liver regeneration is a delicately regulated, self‐enforcing process that involves multiple temporally activated or inhibited pathways.^[^ ^1^ ^]^ Recently, phase separation has been considered as a potential ubiquitous process regulating cellular signal with high specificity and precise spatiotemporal control.^[^ ^15^ ^]^ However, the mechanism by which phase separation regulates liver regeneration remains unclear. Here, we demonstrated that MSL1 played a critical role in liver regeneration. MSL1 formed condensates with STAT3 or H4 by phase separation to enrich Ac‐CoA (a sole donor of acetyl groups), thus promoting the acetylation of STAT3 and H4, further promoting transcriptional activation of STAT3 and increasing expression of the cell cycle‐related genes, respectively. Moreover, Ac‐CoA promoted phase separation of MSL1 both in cells and in vitro, and that pharmacological inhibitor GS‐0976 treatment increased hepatic Ac‐CoA levels, thus promoting liver regeneration after PH in aged mice. Thus, we identified phase separation as an important mechanism by which MSL1 effectively was involved in the acetylation to promote liver regeneration.

MSL1‐deleted mice exhibited a dramatic reduction in both BrdU‐ and Ki67‐positive cells in the livers and liver‐to‐body weight ratio after PH. These findings showed that MSL1 was essential for hepatocytes proliferation after PH. STAT3 activation by macrophage‐derived IL‐6 after PH played an important role in liver regeneration.^[^ ^29^ ^]^ In addition to its phosphorylation, the acetylation of STAT3 also played a role in promoting its transcriptional activation.^[^ ^11^, ^12^ ^]^ MSL1 deletion reduced STAT3 acetylation and phosphorylation levels in mice livers after PH and primary hepatocytes after IL‐6 treatment. Interestingly, the decreased phosphorylation of STAT3 after MSL1 deletion was independent of the IL‐6‐JAK2 pathway, indicating that acetylation of STAT3 might affect its phosphorylation, which is consistent with the previous report that acetylation of STAT3 directly regulates its phosphorylation.^[^ ^11^ ^]^ STAT3 acetylation enhances its dimer stability,^[^ ^12^ ^]^ thereby might inhibiting phosphatase binding to STAT3, in turn increasing its phosphorylation levels, but the specific mechanism remains to be further explored. MSL1 is primarily involved in the acetylation of H4K16.^[^ ^5^ ^]^ Previous study has showed that the activities of multiple histone deacetylases were increased and the level of histone acetylation was decreased after PH.^[^ ^38^ ^]^ However, we found H4K16ac level was dramatically increased in WT mice after PH, which might be due to the specific activation of MSL complex after PH.

IDR domain or CC domain mediate the oligomerization and multivalent interactions, which are required for phase‐separation.^[^ ^16^, ^33^ ^]^ MSL1 contains multiple IDRs and one CC domain. Our data showed that MSL1 depended on IDR3 and CC domain for phase separation. Ac‐CoA is involved in protein acetylation as the substrate of acetyltransferase,^[^ ^23^ ^]^ and Ac‐CoA has the structure partially similar to ATP. ATP can be enriched as a substrate in cGAS‐DNA condensates,^[^ ^20^ ^]^ and ATP has been reported to promote phase separation of DEAD‐box ATPases,^[^ ^18^ ^]^ but another study has indicated that ATP inhibits phase separation.^[^ ^19^ ^]^ Our results showed that Ac‐CoA facilitated the phase separation of MSL1 in cells and in vitro, and in vitro sedimentation assay showed that Ac‐CoA was enriched in MSL1 condensates. Since Ac‐CoA is difficult to track in cells, whether Ac‐CoA can be enriched in MSL1 condensates in cells remains unclear. Although Ac‐CoA can diffuse freely in the nucleus and cytoplasm, there is still a difference in the spatial distribution.^[^ ^37^ ^]^ Our results showed that phase separation might be one of the mechanisms causing the spatial distribution difference of Ac‐CoA. Previous studies have indicated that STAT3 and histone forms condensates in cells and in vitro.^[^ ^34^, ^35^ ^]^ However, our results showed that STAT3 or H4 did not form condensates when it was overexpressed in HEK 293T cells or purified in vitro, but STAT3 or H4 formed condensates with MSL1 and promote its acetylation. This pattern is similar to that of EZH2, which compartmentizes STAT3 through phase separation, thus promoting STAT3 phosphorylation.^[^ ^39^ ^]^

Epigenetic silencing plays an important role in aging‐induced liver regeneration weakness.^[^ ^40^ ^]^ Ac‐CoA is widely involved in epigenetic modification regulation, and increasing Ac‐CoA level can delay aging by increasing histone acetylation.^[^ ^25^ ^]^ In addition, high‐level Ac‐CoA signifies that metabolic resources are sufficient for growth and proliferation.^[^ ^26^ ^]^ Interestingly, in budding yeast, acetate‐derived Ac‐CoA reduced telomere silencing and accelerated cell senescence by increasing H4K16ac levels.^[^ ^41^ ^]^ We found that GS‐0976 treatment increased hepatic Ac‐CoA levels, thus enhancing the acetylation of STAT3 and H4, finally promoting liver regeneration after PH in aged mice. Ac‐CoA maintains mitochondrial function^[^ ^25^ ^]^ and provides substrate for ATP production, and normal mitochondrial function and ATP production are necessary for liver regeneration.^[^ ^42^, ^43^ ^]^ Therefore, Ac‐CoA might promote liver regeneration via multiple pathways.

In conclusion, this study indicates that MSL1 promotes the liver regeneration in mice after PH. MSL1 enhances the acetylation of STAT3 and H4 by forming condensates with STAT3 or H4, and the formed condensates enrich Ac‐CoA, thus promoting the priming of hepatocyte and cell cycle progression. The increased Ac‐CoA level in the liver can promote liver regeneration in aged mice. Our results suggest that the regulation of phase separation of MSL1 and Ac‐CoA level may be a novel therapeutic strategy for acute liver diseases and liver transplantation.

## 4 Experimental Section

### Animal Model

The floxed MSL1 mice (Cyagen Biosciences, China) were crossed with Albumin‐Cre recombinase transgenic mice (Model Animal Research Center of Nanjing University, China) to generate MSL1 LKO mice with its open reading frame of MSL1 deleted. The wild‐type (WT) littermates (Albumin‐Cre negative, MSL1 flox/flox) were used as the control. C57BL/6J 3‐month‐old WT (young) mice and 14‐month‐old WT (aged) mice were obtained from Huazhong Agricultural University Laboratory Animal Centre. All the mice used in the experiment were male. GS‐0976 (MCE, HY‐16901) was dissolved in vehicle (veh, saline solution containing 1% Tween 80 and 0.5% methylcellulose) and administered to mice by gavage at a dose of 10 mg kg^−1^ once a day for 28 days. pcDNA3.1+‐GFP or pcDNA3.1+‐GFP‐MSL1 plasmids was delivered into mice via Hydrodynamic tail vein injection.^[^ ^44^ ^]^ Mice were rapidly injected (5–8 s) with 30 µg endotoxin‐free pcDNA3.1+‐GFP or pcDNA3.1+‐GFP‐MSL1 plasmids diluted in Ringer solution in a total volume equal to 10% of their body weight. The mice were sacrificed 48 h after injection. All the mice were housed under a 12:12‐h light/dark cycle at controlled temperature. The PH was performed as follows: the two‐thirds of the liver was surgically removed, and the left lateral, caudate, and median lobes were completely excised with the gallbladder left intact, as described previously.^[^ ^45^ ^]^ BrdU (Sigma–Aldrich, B5002) was injected at the dose of 50 mg kg^−1^ 2 h before sacrifice. Tissue and serum were collected at the end of the experiments. All the animal experimental procedures followed the Huazhong Agricultural University Guidelines for the Care and Use of Laboratory Animals (XYXK(Hubei) 2020‐0084).

### Isolation of Primary Mouse Hepatocytes and Cell Sorting

Primary mouse hepatocytes were isolated by the previously reported method with minor modification.^[^ ^46^ ^]^ Briefly, the WT and LKO mice were anesthetized with Avertin (Sigma–Aldrich, T48402, 240 mg kg^−1^) by intraperitoneal injection, and the hepatic portal vein was cannulated. The liver was perfused at 6 mL min^−1^ with pre‐warmed perfusion medium containing EGTA for 8 min. After the first wash, a second perfusion was performed with prewarmed digestion medium containing collagenase IV at 4 mL min^−1^ for 5 min. Hepatocytes were filtered through a 100 mm filter membrane, separated by centrifugation at low speed (50 g, 5 min), and purified with Percoll (Sigma–Aldrich, P4937) gradient centrifugation. For cell sorting, Isolated hepatocytes sent to FACS, sorting was performed at 4 °C and samples were collected in growth media (high‐glucose DMEM supplemented with 10% fetal bovine serum (FBS), and 1% penicillin/streptomycin). Only GFP‐positive cells were sorted with Cytoflex SRT (Beckman Coulter, Pasadena, CA, USA) using a 100‐mm nozzle. Sorted cells were immediately centrifuged and resuspended in RNAiso Plus (Takara, 9018) for total RNA isolation or RIPA lysis buffer (Beyotime, P0013B) for immunoblotting assay.

### Plasmid Construction

To construct eukaryotic expression plasmid, MSL1, STAT3, and H4 were individually amplified from mouse liver cDNAs. MSL1 was inserted into pcDNA3.1+ with or without Myc‐tag or pEGFP‐N1 vector, and STAT3 and H4 were inserted into PCS‐mCherry or pcDNA3.1+ with Flag‐tag vector, using one‐step cloning method (Vazyme, 115‐01). The plasmids encoding GFP‐MSL1‐ΔIDR1, GFP‐MSL1‐ΔIDR2, GFP‐MSL1‐ΔCC, GFP‐MSL1‐ΔIDR3, and GFP‐MSL1‐ΔIDR4 were amplified from pEGFP‐N1‐GFP‐MSL1 using one‐step cloning method.

To construct protein purification plasmids, the sequences of MSL1, STAT3, H4, GFP, GFP‐MSL1, mCherry‐STAT3, and H4‐mCherry were inserted into pET‐28a vector using one step cloning method for protein purification from *E. coli*.

### Cell Culture, Transient Transfection, and Treatment

Cells were grown in high‐glucose DMEM (Hyclone, Logan, UT, USA) containing 10% FBS (Thermo Fisher Scientific, Waltham, USA) and 1% penicillin‐streptomycin (Hyclone, sv30010). Cells were cultured in a carbon dioxide incubator (Thermo Fisher Scientific) with 5% CO_2_ at 37 °C overnight. The following day, the cells were transient transfected with plasmids using Lipofectamine 2000 (Invitrogen, Carlsbad, CA) according to the manufacturer's instructions. HEK 293T cells, Hep1‐6 cells, and primary mouse hepatocytes respectively treated with human IL‐6 (MCE, HY‐P7044) for 30 min for immunoblot analysis. Primary mouse hepatocytes were treated with DMSO (vehicle), CMS‐121 (1 × 10^−3^ m; MCE, HY‐135981), and SB 204 990 (10 × 10^−3^ m; MCE, HY‐104032) for 24 h, respectively, for immunofluorescent and immunoblot analysis.

### Serum ALT/AST and IL‐6 Level

Serum was collected from blood by centrifugation (1000 g) for 10 min at 4 °C. The levels of serum ALT, AST, and IL‐6 were measured using ALT/AST determination kit (Nanjing Jiancheng Bioengineering Institute, China) and IL‐6 ELISA kit (Pierce Biotechnology) according to the manufacturer's instructions.

### H&E and Immunohistochemical Staining

Livers were fixed in 4% PBS‐buffered formalin, dehydrated, and embedded in paraffin. Liver sections were stained with H&E (Beyotime, C0105S) or subjected to immunohistochemical staining. Immunohistochemical staining was performed to detect targets using antibodies anti‐Ki67 (Thermo Fisher Scientific, PA5‐19462) and anti‐BrdU (Thermo Fisher Scientific, MA3‐071), horseradish peroxidase‐conjugated goat anti‐mouse (Proteintech, PR30011) and goat anti‐rabbit (Proteintech, PR30012) antibodies. Blocking and chromogenic detection were performed using the DAKO Envision System with DAB substrate (DAKO, Denmark) according to the manufacturer's protocol. BrdU‐ and Ki67‐positive cells were counted using ImageJ.

### Immunofluorescence Staining

Cells were cultured on coverslips and fixed with 4% PFA for 10 min at room temperature. Cells were incubated in PBS buffer containing 0.1% Triton X‐100 for 20 min and blocked (10% goat serum in PBS) for 60 min at room temperature. Cells were incubated with primary antibody MSL1 (Novus, #90 506) overnight at 4 °C, washed three times with PBS, and incubated with secondary antibody Alexa Fluor 555 goat anti‐rabbit IgG (Invitrogen, A‐21428) for 60 min at room temperature. Cells were counterstained with DAPI (Abcam, ab104139), observed under a Nikon confocal microscope with 100× oil objective, and analyzed by NIS software with fixed parameters.

### Immunoblotting and Coimmunoprecipitation

Cells and liver tissues were lysed in RIPA lysis buffer containing protease inhibitors (Sigma–Aldrich, P8340), phosphatase inhibitors (Sigma–Aldrich, P2850), and deacetylase inhibitors (Beyotime, P1112) according to the manufacturer's instructions. Lysates were denatured by heating for 10 min at 95 °C and loaded onto SDS–PAGE gel. Afterwards, the gel was transferred to polyvinylidene difluoride membranes (Millipore, IPVH00010), and the membranes were blocked with 5% skimmed milk and incubated with the corresponding primary antibody overnight at 4 °C. Subsequently, the membranes were incubated with horseradish peroxidase‐conjugated secondary antibody at room temperature for 1 h. Finally, the membranes were visualized with enhanced chemiluminescence (Abbkine, BMU102‐CN). This study used the antibodies against the following proteins: Phospho‐STAT3 (Tyr705) (#9145), acetyl‐STAT3 (Lys685) (#2523), STAT3 (#9139), Phospho‐Jak2 (Tyr1007/1008) (#3771), acetyl‐histone H4 (Lys16) (#13 534) from Cell Signaling Technology (Danvers, MA, USA); MSL1 (#90 506) from Novus; GADPH (#A19056), anti‐Myc (#AE070), anti‐FLAG (#AE092) from Abclonal (Wuhan, China); histone H4 (16047‐1‐AP), histone H3 (68345‐1‐Ig) from Proteintech (Wuhan, China). For coimmunoprecipitation experiments, cells were lysed in immunoprecipitation lysis buffer (Beyotime, P0013) containing protease inhibitors. The resultant lysates were added with 1 mg indicated antibody and incubated with protein A/G magnetic beads (MCE, HY‐K0202) at 4 °C overnight. The magnetic bead‐enriched immunocomplexes were separated on SDS–PAGE, transferred to PVDF membranes for immunoblotting assay.

### RNA Isolation and Quantitative Real‐Time PCR (qRT‐PCR)

Total RNA was extracted from frozen samples with the RNAiso Plus according to the manufacturer's protocol. Subsequently, the first‐strand cDNA was synthesized using the PrimeScript RT Reagent Kit with gDNA Eraser (Takara, RR047A). qRT‐PCR was performed with the MonAmp SYBR Green qPCR Mix (MQ10201S, Low ROX) using QuantStudio 3 Real‐Time PCR Instrument (Applied Biosystems, USA). The relative expression levels of mRNA were calculated using the 2^−ΔΔCt^ method. The primer sequences were listed in Table S1 (Supporting Information).

### Fluorescence Recovery After Photo Bleach (FRAP)

HEK 293T cells were cultured in Laser confocal dish and transfected with plasmid for fluorescence recovery after photo bleach (FRAP) in live cells, as described above. Cell condensates were fully or partially photobleached with 70% laser power for 2 s using a 488 and/or 561‐nm laser, and time‐lapse images were captured within 3 min after bleaching with 5 s interval. In vitro condensates were photobleached with 70% laser power for 2 s using 488‐nm lasers, and time‐lapse images were captured within 2 min after bleaching with 5 s interval. GraphPad Prism was used to plot and analyze the FRAP results.

### Living Cell Imaging

HEK293T cells were grown on laser confocal dish, transiently transfected with GFP‐MSL1, and imaged on an upright Nikon confocal microscope with a 100× oil objective and FITC filter sets for GFP. Afterwards, transfected HEK293T cells were grown on laser confocal dish containing 1 mL of high‐glucose DMEM and imaged every 15 s. Before the second imaging, 1 mL of 6% 1,6‐hex or 2,5‐hex was added on the dish within 10 s for further live cell imaging.

### Chromatin Immunoprecipitation (ChIP) Assay

ChIP assay was performed using the ChIP assay kit (Beyotime, P2080S) according to the user manual. In brief, mouse livers were incubated with formaldehyde (1%) for 10 min at 37 °C to cross‐link the nuclear proteins to DNA. Subsequently, livers were rinsed with ice‐cold PBS and lysed in SDS lysis buffer followed by sonication and immunoprecipitation with the antibody against H4K16ac (Cell Signaling Technology, #13 534). The reaction with additional IgG was used as a negative control, and the reaction without additional anti‐H4K16ac and IgG was used as mock control. The captured chromatin was eluted and un‐crosslinked, and the DNA was recovered. The ChIP‐isolated DNA was subjected to PCR and qRT‐PCR analyses using the primer pair spanning Cyclin A2, Cyclin B1, and Cyclin D1 promoter region. The primer sequences were listed in Table S2 (Supporting Information).

### Ac‐CoA Content Measurement

Ac‐CoA content was measured using the PicoProbe Ac‐CoA assay kit (Abcam, ab87546) according to manufacturer's instructions. Briefly, after deproteinization using the perchloric acid, the CoA‐SH Quencher and Quencher remover were added into the samples to correct the background generated by free CoA‐SH and succ‐CoA. The samples were then diluted with the reaction mixture, and the fluorescence of samples was measured using a multifunctional microplate reader (EnVision PE, USA) with the parameters settings of *λ*_ex_ = 535 nm and *λ*_em_ = 587 nm. The Ac‐CoA standard curve was constructed in the range of 0–100 × 10^−12^ m with the correlation coefficient of ≥0.99.

### Protein Expression and Purification

PET28a plasmids containing His‐tagged genes were transformed into *E. coli* BL21 cells. Cells were grown in LB medium containing 30 µg mL^−1^ Kanamycin at 37 °C until optical density (OD) reached 0.6, and induced with 0.5 × 10^−3^ m isopropyl‐b‐d‐thiogalactopyranoside at 16 °C for 20 h. Cells were collected by centrifugation and lysed by high‐pressure homogenizer in the buffer (pH 7.8) containing 20 × 10^−3^ m Tris‐HCl, 300 × 10^−3^ m NaCl, 20 × 10^−3^ m imidazole, and 0.2 × 10^−3^ m PMSF, and cell debris was removed by centrifugation at 10 000 g for 30 min. Then, lysate was loaded onto a 1‐mL HiTrap HP chelating column (Cytiva, 17 524 701). The column was washed, and the protein was eluted with the buffer (pH 7.8) containing 20 × 10^−3^ m Tri‐HCl,500 × 10^−3^ m NaCl, and 250 × 10^−3^ m imidazole. Afterwards, the protein was concentrated with Amicon Ultra Centrifugal Filters (Millipore), and protein concentrations were measured by ultraviolet absorbance at 280 nm. The size and purity of the purified proteins were monitored by SDS–PAGE with Coomassie blue staining.

### In Vitro Phase Separation Assays

Purified proteins were diluted to various concentrations in buffer (pH 7.8) containing 20 × 10^−3^ m HEPES and the indicated NaCl, Ac‐CoA, PEG‐8000, or 1,6‐hex concentrations. Protein solution (10 µL) was loaded onto a glass slide, covered with a coverslip, and imaged using a Nikon confocal microscope with 100× oil objective.

For the sedimentation assay, samples were centrifugated at 15 000 g for 10 min on a table‐top temperature controlled microcentrifuge. Supernatant and pellets were separated and put into two tubes immediately after centrifugation. The pellet fraction was resuspended with the same buffer to an equal volume as the supernatant fraction. Proteins from both fractions were analyzed by SDS‐PAGE with Coomassie blue staining. Band intensity was quantified using the ImageJ

### Statistical Analysis

All experiments were repeated at least three times. Statistical analyses were performed using the GraphPad Prism 6 software. The data were expressed as means ± SD. Student's two‐tailed *t*‐test (unpaired) was used to determine statistical significance differences between groups. Statistical significance was presented at the level of ^*^ *p* < 0.05, ^**^ *p* < 0.01, ^***^ *p* < 0.001, and ^****^ *p* < 0.0001.

## Conflict of Interest

The authors declare no conflict of interest.

## Author contributions

L.Z. and Y.H. conceived and designed the study; Y.H. provided the experimental data. Y.H., S.W. and S.L. performed the cell and animal experiments; Y.H., S.L. and D.Q. provided assistance in molecular assays; Y.H., S.L., D.Q. and Z.L. discussed and drafted the manuscript; L.Z., L.W. and X.C. reviewed the manuscript; L.Z. organized the data and wrote the manuscript.

## Supporting information

Supporting Information

## Data Availability

The data that support the findings of this study are available from the corresponding author upon reasonable request.
