# Supplementary material for: MSL1 Promotes Liver Regeneration by Driving Phase Separation of STAT3 and Histone H4 and Enhancing Their Acetylation
Source: Adv Sci (Weinh). 2023 Jun 6;10(23):2301094. doi: 10.1002/advs.202301094 (PMC10427353; doi:10.1002/advs.202301094)
Supplement: Supplementary file 1 — Supporting Information [file ADVS-10-2301094-s001.pdf]

ADVANCED SCIENCE
Open Access

Supporting Information

for *Adv. Sci.*, DOI 10.1002/advs.202301094

MSL1 Promotes Liver Regeneration by Driving Phase Separation of STAT3 and Histone H4 and Enhancing Their Acetylation

*Yucheng He, Shichao Wang, Shenghui Liu, Dan Qin, Zhangmei Liu, Liqiang Wang, Xiangmei Chen and Lisheng Zhang**

Supporting Information

MSL1 promotes liver regeneration by driving phase separation of STAT3 and Histone H4 and enhancing their acetylation

*Yucheng He, Shichao Wang, Shenghui Liu, Dan Qin, Zhangmei Liu, Liqiang Wang, Xiangmei Chen and Lisheng Zhang**

Supplementary Figures and Figure legends

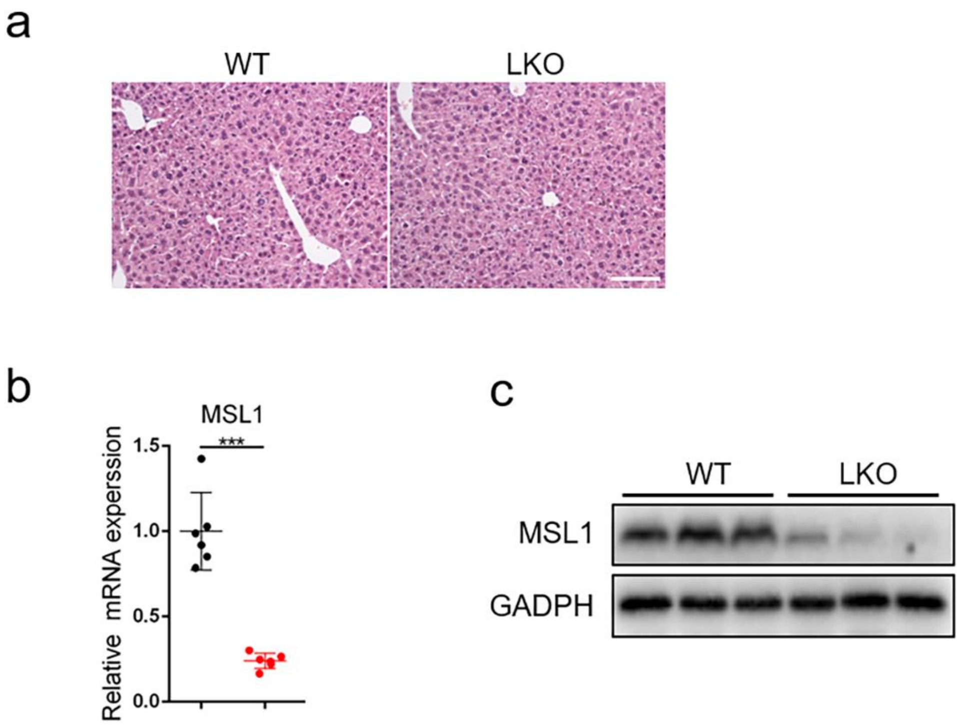

Figure S1. Related to Figure 1. a) H&E staining of liver tissue sections of WT and LKO mice. Scale bar, 50 μm. b) qRT-PCR analysis of MSL1 mRNA expression in WT and LKO mouse livers (n = 6 mice/group). c) Immunoblot analysis of liver tissue lysates from WT and LKO mice using the indicated antibodies (n = 3 mice/group). The data were expressed as means ± SD. Significant difference was presented at the level of ***p < 0.001 by two-tailed Student's t-test.

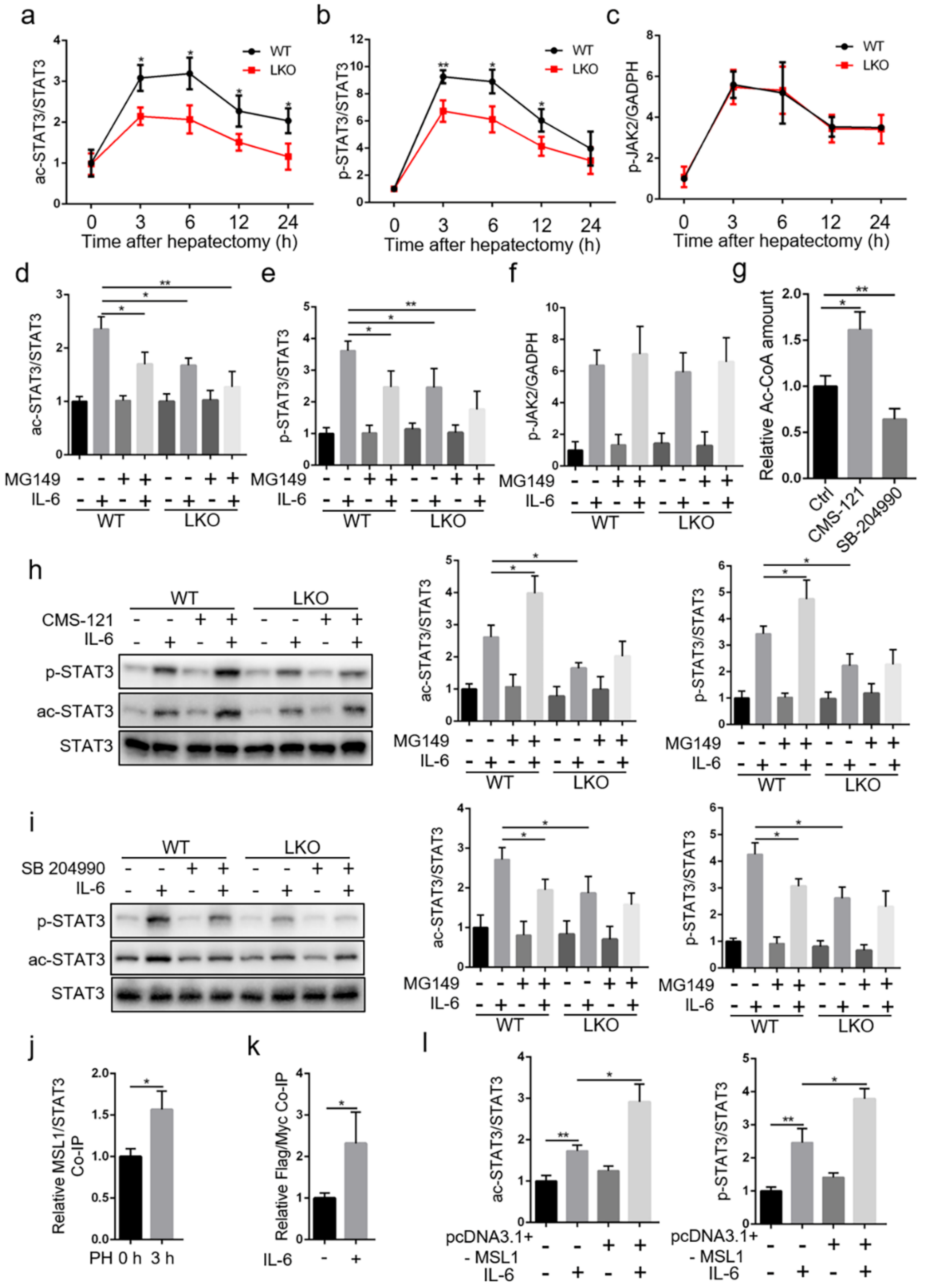

Figure S2. Related to Figure 2. a-c) Quantification of protein levels of ac-STAT3 (a), p-STAT3 (b) and p-JAK2 (c) as in (Figure 2a) by densitometric analysis and normalization versus loading control (n = 3). d-f) Quantification of protein levels of ac-STAT3 (d), p-STAT3 (e) and p-JAK2 (f) as in (Figure 2b) by densitometric analysis and

normalization versus loading control (n = 3). g) Primary mouse hepatocytes treatment with 1 μM CMS-121 or 10 μM SB 204990 for 24 h and quantification of the Ac-CoA concentration (n = 3). h,i) Immunoblot analyses were performed on primary mouse hepatocytes prepared from WT and LKO mice, and treatment with 1 μM CMS-121 (h)/10 μM SB-204990 (i) for 24 h and/or 10 ng mL^{-1} IL-6 for 30 mins using the indicated antibodies. Right panels show the quantification of protein levels of ac-STAT3 and p-STAT3 by densitometric analysis and normalization versus loading control (n = 3). j) Quantification of protein levels of MSL1 as in (Figure 2f) by densitometric analysis and normalization versus immunoprecipitated STAT3 (n = 3). k) Quantification of protein levels of Flag-H4 as in (Figure 2g) by densitometric analysis and normalization versus immunoprecipitated Myc-MSL1 (n = 3). l) Quantification of protein levels of ac-STAT3 and p-STAT3 as in (Figure 2h) by densitometric analysis and normalization versus loading control (n = 3). The data were expressed as means ± SD. Significant difference is presented at the levels of *$p < 0.05$ and **$p < 0.05$ by two-tailed Student's t-test.

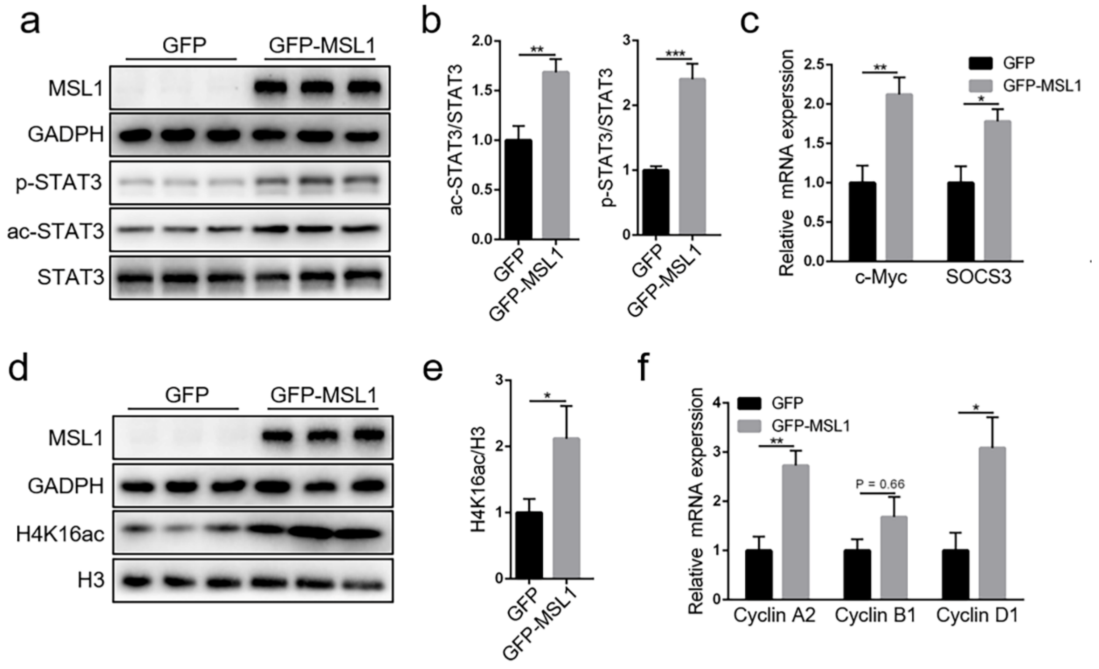

Figure S3. Overexpression of MSL1 in the hepatocytes promoted STAT3 and H4K16 acetylation. a) Immunoblotting analysis of GFP-positive primary mouse hepatocytes sorted by FACS at 3 h post-PH in mice using the indicated antibodies. b) Quantification of protein levels of ac-STAT3 and p-STAT3 as in (Figure S3a) by densitometric analysis and normalization versus loading control ($n = 3$). c) qRT-PCR analysis of c-Myc and SOCS3 mRNA expression in GFP-positive primary mouse hepatocytes sorted by FACS at 3 h post-PH in mice ($n = 3$). d) Immunoblotting analysis of GFP-positive primary mouse hepatocytes sorted by FACS at 36 h post-PH in mice using the indicated antibodies. e) Quantification of protein levels of H4K16ac as in (Figure S3d) by densitometric analysis and normalization versus loading control ($n = 3$). f) qRT-PCR analysis of Cyclin A2, Cyclin B1 and Cyclin D1 mRNA expression in GFP-positive primary mouse hepatocytes sorted by FACS at 36 h post-PH in mice ($n = 3$). The data were expressed as means ± SD. Significant difference was presented at the level of $*p < 0.05$, $**p < 0.01$, and $***p < 0.001$ by two-tailed Student's t-test.

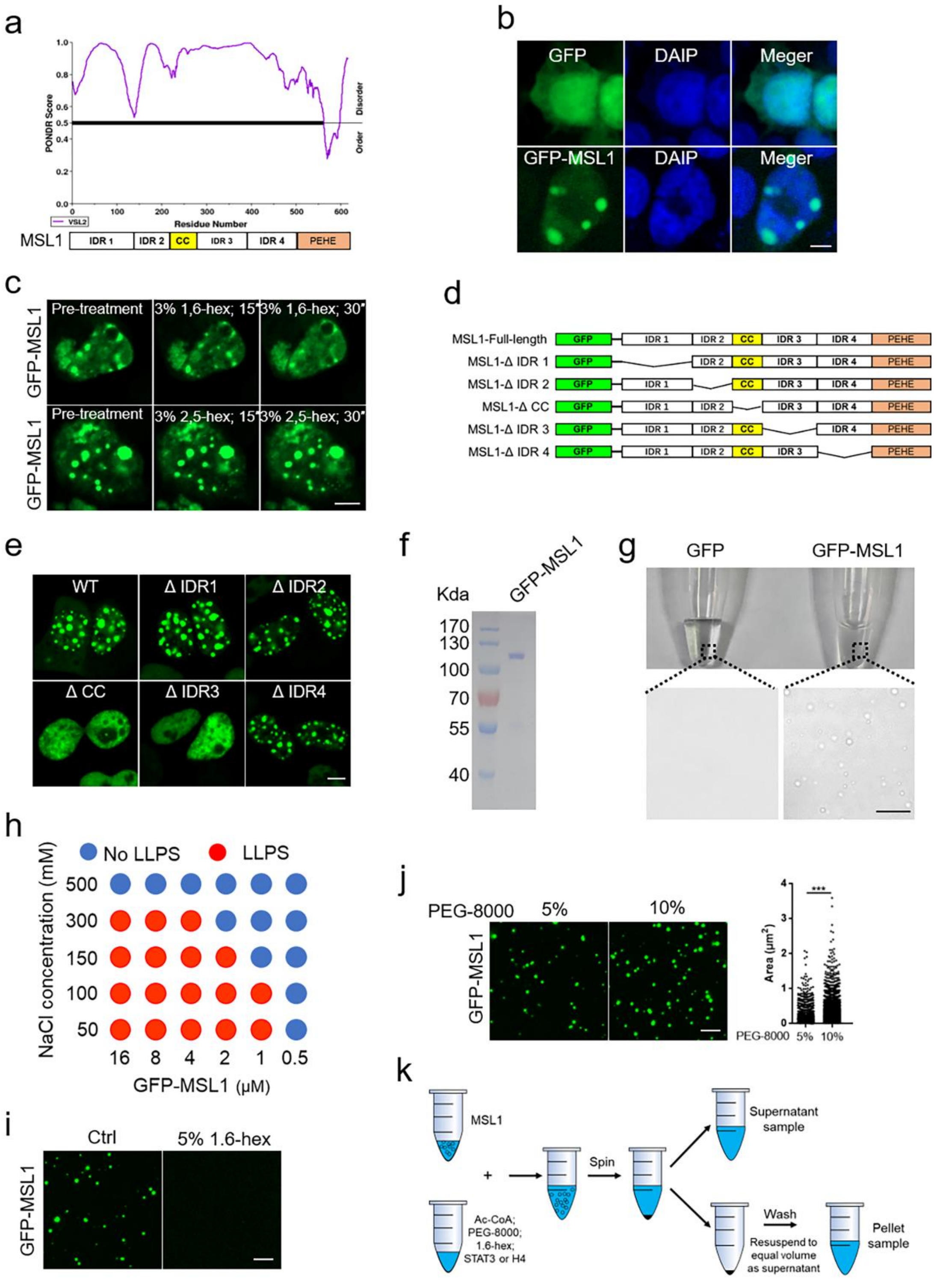

Figure S4. related to Figure 4. a) Graphs of IDR of MSL1 plotted by PONDR VSL2. b) Confocal images of fixed HEK293T cells transfected with GFP-MSL1 or GFP for 24 h. Scale bar, 5 μm. c) Confocal images of GFP-MSL1-transfected HEK293T cells before and after 1,6-hex or 2,5-hex treatment at indicated time points. Scale bar, 5 μm. d) Diagram of full-length and truncated GFP-MSL1 proteins. e) Confocal images of

HEK 293T cells transfected with full-length or truncated GFP-MSL1 plasmids. Scale bar, 5 μm. f) SDS-PAGE assay and Coomassie blue staining of GFP-MSL1 protein purified from *E. coil*. g) Images of GFP-MSL1 and GFP taken at room temperature. Scale bar, 10 μm. h) Diagrams of GFP-MSL1 phase separation at various concentrations of salt (NaCl) and proteins. i) Images of GFP-MSL1 (8 μM) condensates at room temperature with or without 5% 1,6-hex treatment. Scale bar, 5 μm. j) Images of GFP-MSL1 (8 μM) condensates at room temperature with 5% or 10% PEG-8000 treatments. n = 3 fields (100 × 100 μm^2). Scale bar, 5 μm. k) Schematic diagram of sedimentation assay to separate the condensed liquid phase and the aqueous phase with additional MSL1, Ac-CoA, PEG-8000, 1.6-hex, STAT3 or H4. The data were expressed as means ± SD. Significant difference was presented at the level of ***$p < 0.001$ by two-tailed Student's t-test.

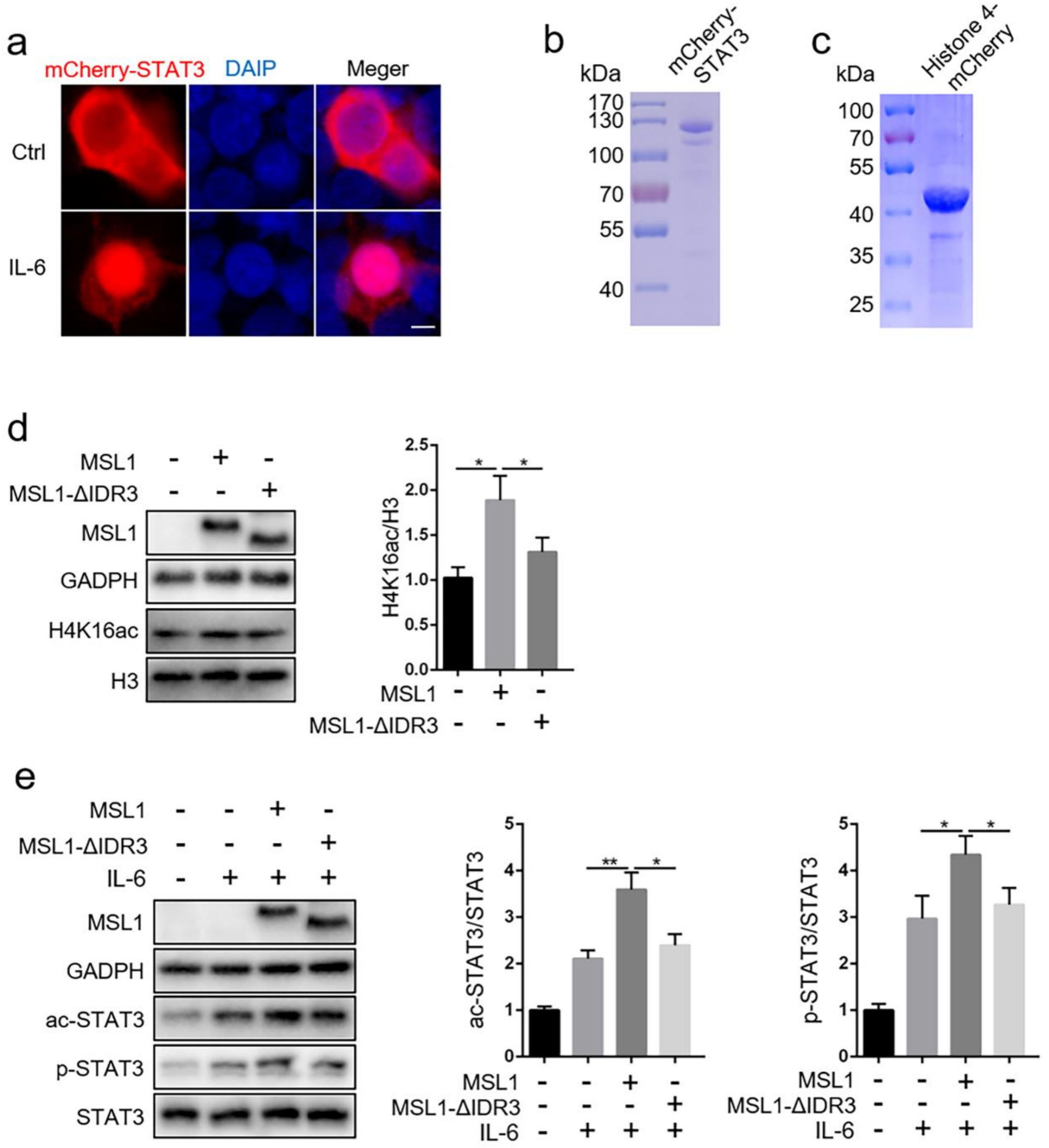

Figure S5. Related to Figure 5. a) Confocal images of fixed HEK293T cells transfected with mCherry-STAT3-encoding plasmids for 24 h without or with 10 ng mL^{-1} IL-6 for 30 min. Scale bar, 5 μm. b,c) SDS-PAGE assay and Coomassie blue staining of mCherry-STAT3 (b) and Histone 4-mCherry (c) purified from *E. coil*. d) Immunoblot analysis of Hep1-6 cells transfected with MSL1 or MSL1-ΔIDR3 plasmids for 24 h using the indicated antibodies. Right panel show the quantification of protein levels of H4K16ac by densitometric analysis and normalization versus loading control (n = 3). e) Immunoblot analysis of Hep1-6 cells transfected with MSL1 or MSL1-ΔIDR3 plasmids for 24 h with or without 30-min IL-6 treatment (10 ng mL^{-1}) using the indicated antibodies. Right panels show the quantification of protein levels of ac-

STAT3 and p-STAT3 by densitometric analysis and normalization versus loading control ($n = 3$). The data were expressed as means ± SD Significant difference is presented at the levels of *$p < 0.05$ and **$p < 0.01$ by two-tailed Student’s t-test.

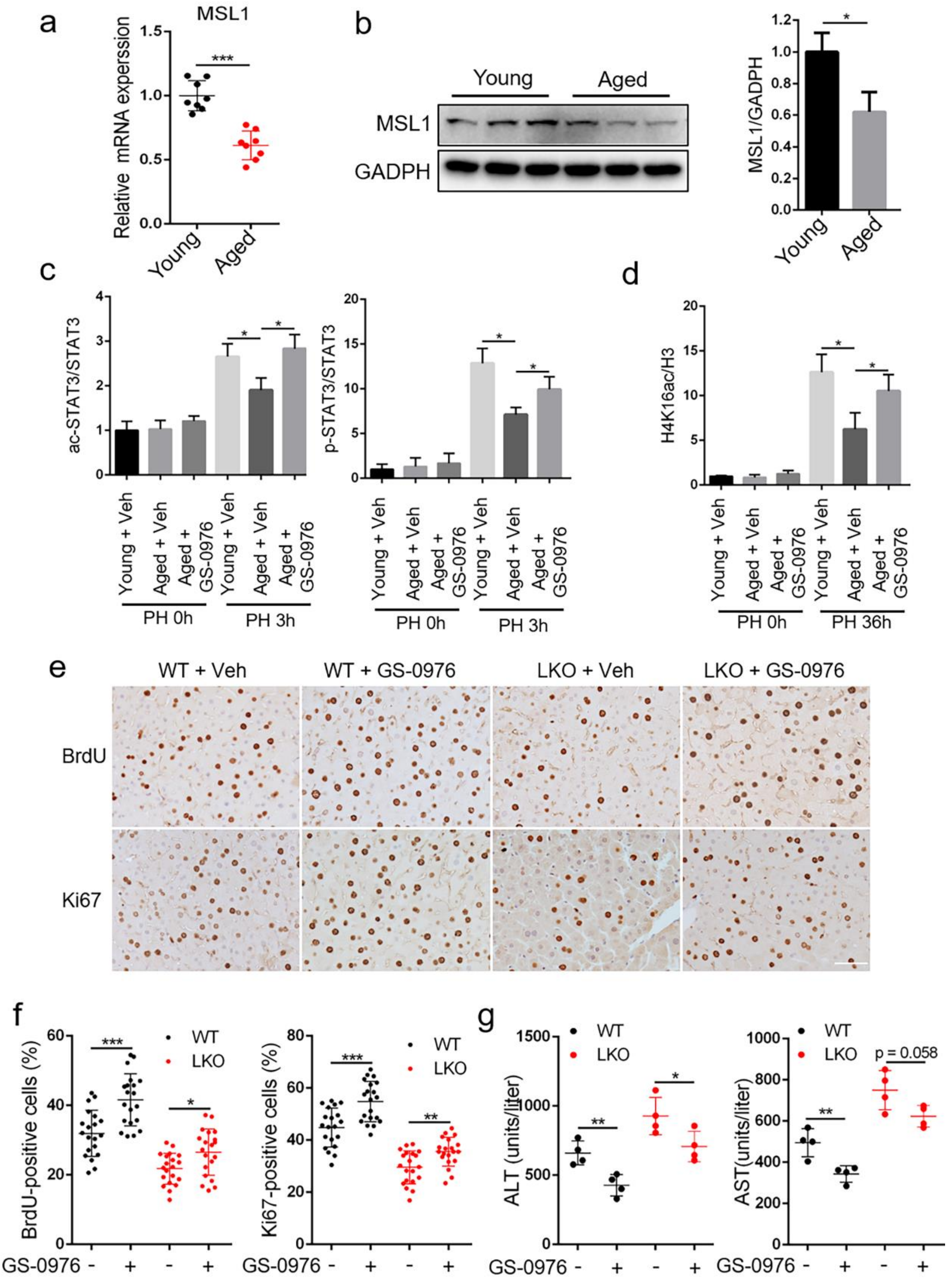

Figure S6. Related to Figure 6. a) qRT-PCR analysis of MSL1 in aged and young mouse livers (n = 8 mice/group). b) Immunoblot analysis of liver tissues lysates from young and aged mice using the indicated antibodies. c) Quantification of protein levels of ac-STAT3 and p-STAT3 as in (Figure 6f) by densitometric analysis and normalization versus loading control (n = 3). d) Quantification of protein levels of H4K16ac as in

(Figure 6g) by densitometric analysis and normalization versus loading control ($n = 3$). e) BrdU and Ki67 staining of liver tissues from WT and LKO mice treatment with or without GS-0976 at 36 h after PH. Scale bar, 50 μm. f) BrdU- and Ki67-positive cell count at 36 h after PH, $n = 4$ mice/group, 5 fields (215×285 μm^2) quantified/animal. g) Serum ALT and AST levels in WT and LKO mice treatment with or without GS-0976 at 36 h after PH ($n = 4$ mice/group). The data were expressed as means ± SD. Significant difference was presented at the level of ***$p < 0.001$ by two-tailed Student's t-test.

Supplementary Tables

Table S1. Primers used in qRT-PCR

Name	Location	Sequence (5′ to 3′)	nt (bp)
MSL1	NM_028722.3	F: TTTCTCATGTGGCCGGAGTG<br>R: GGGGTTTCTTGGCTCCTCAA	204
SOCS3	NM_007707.3	F: CCCCAAGAGAGCTTACTACA<br>R: TTTCTCATAGGAGTCCAGGT	139
c-Myc	NM_010849.4	F: TCCACTCACCAGCACAACTACG<br>R: GCTGATCTGCTTCAGGACCCT	105
Cyclin A2	NM_009828.3	F: TGAAGAGGCAGCCAGACATCAC<br>R: AGCCAAATGCAGGGTCTCAT	107
Cyclin B1	NM_172301.3	F: AAGGTGCCTGTGTGTGAACC<br>R: AGATACTCTTCTGCAGGCGC	164
Cyclin D1	NM_001379248.1	F: GCGTACCCTGACACCAATCTC<br>R: CTCCTCTTCGCACTTCTGCTC	183
36B4	NM_007475.5	F: TGGAGACAAGGTGGGAGCC<br>R: CACAGACAATGCCAGGACGC	271

Table S2. Primers used in Chip-PCR and Chip-qPCR

Name	Sequence (5′ to 3′)
Cyclin A2-F	GGGACAGCATTATGAGACCCTG
Cyclin A2-R	CCCCGTCCTGGGTACACTAC
Cyclin B1-F	GTGAGCACTGCACGCCATGC
Cyclin B1-R	GAATGCGTTTCCTGGGCGATC
Cyclin D1-F	TCTTCCTTGGCTTGCGTGTG
Cyclin D1-R	GAGACCTGTGGAGGTGGGG
